# Risk factors for lower limb lymphedema after lymph node dissection in patients with ovarian and uterine carcinoma

**DOI:** 10.1186/1471-2407-9-47

**Published:** 2009-02-05

**Authors:** Harue Tada, Satoshi Teramukai, Masanori Fukushima, Hiroshi Sasaki

**Affiliations:** 1 Department of Clinical Trial Design and Management, Translational Research Center, Kyoto University Hospital, 54 Shogoin Kawahara-cho, Sakyo-ku, Kyoto, 606-8507, Japan; 2 Department of Obstetrics and Gynecology, The Jikei University School of Medicine Kashiwa Hospital, 163-1 Kashiwashita, Kashiwa, Chiba, 277-8567, Japan

## Abstract

**Background:**

Lymph node dissection has proven prognostic benefits for patients with ovarian or uterine carcinoma; however, one of the complications associated with this procedure is lymphedema. We aimed to identify the factors that are associated with the occurrence of lymphedema after lymph node dissection for the treatment of ovarian or uterine carcinoma.

**Methods:**

A total of 694 patients with histologically confirmed ovarian (135 patients) or uterine cancer (258 with cervical cancer, 301 with endometrial cancer) who underwent lymph node dissection were studied retrospectively. Logistic regression analyses were used to identify the risk factors associated with occurrence of lymphedema.

**Results:**

Among ovarian and uterine cancer patients who underwent pelvic lymph node dissection, post-operative radiotherapy (odds ratio: 1.79; 95% confidence interval: 1.20–2.67; p = 0.006) was statistically significantly associated with occurrence of lymphedema.

**Conclusion:**

There was no relationship between any surgical procedure and occurrence of lymphedema among patients undergoing pelvic lymphadenectomy. Our findings are supported by a sound biological rationale because they suggest that limb lymphedema is caused by pelvic lymph node dissection.

## Background

After breast cancer, ovarian and uterine carcinomas are the next most-common gynecological malignancies in Japan [1]. Owing to the recent development of effective chemotherapeutic agents and regimens, some cases of ovarian cancer are now curable with a combination of surgery and chemotherapy, and the survival of patients has continued to improve [2]. For early-stage ovarian cancer, the standard therapy is complete surgical staging followed by abdominal hysterectomy and bilateral salpingo-oophorectomy with omentectomy and selective lymphadenectomy. Uterine cancer is also potentially curable using the following therapeutic approaches: (i) radiation, (ii) surgery, and (iii) chemotherapy [1]. As more effective modes of treatment, such as extended surgical removal and lymph node dissection are implemented, many patients with ovarian or uterine carcinoma have been cured and survivorship has continued to increase [3].

However, an important but sometimes overlooked sequel to these treatments is lymphedema of the lower limbs [4]. This is a chronic, incurable condition, the effects of which include limb swelling and feelings of heaviness, tightness, and pain [5]. Lymphedema can take a psychological toll, with patients experiencing symptoms such as anxiety, depression, and adjustment problems [6]. Consequently, lymphedema can affect the patients' vocational, domestic, social, and sexual lives and adversely affect their quality of life [7]. Hence, prevention of lymphedema has become an important issue in the care of patients who undergo lymphadenectomy.

In previous studies, risk factors for the incidence of lymphedema have been identified, including the removal of pelvic lymph nodes [8,9] subsequent to the diagnosis of malignancy, which may obstruct the flow of lymphatic fluid from the lower limbs [10]. Also, a recent study found that patients who had 10 or more regional lymph nodes removed during the initial surgery appeared to be at a higher risk for developing new symptomatic limb lymphedema [11]. It is unclear which surgical procedures elevate the risk of lower limb lymphedema (LLL) after lymph node dissection for gynecological malignancies. We conducted a retrospective study to identify the risk factors that were associated with occurrence of LLL after lymph node dissection for the treatment of ovarian or uterine carcinoma.

## Methods

A total of 694 patients with histologically proven uterine (258 patients with cervical cancer and 301 with endometrial cancer) or ovarian (135 patients) carcinoma treated with lymph node dissection between January 1997 and December 1998 were enrolled consecutively at 10 Japanese hospitals (The Jikei University School of Medicine Hospital, Sapporo Medical University Hospital, Niigata Cancer Center Hospital, Hyogo Medical Center for Adults, Aichi Cancer Center, Toyama Prefectural Central Hospital, National Kure Medical Center, Shikoku Cancer Center, Nagasaki University, Shinshu University Hospital). Lymphadenectomy was defined as surgery to remove more than twenty lymph nodes. The protocol of this study was approved by the ethics committee at each institution. Informed consent was obtained from each of the patients during their visit, after an explanation of the study and its methods, including collection of data from their past medical records.

Patients who had another active cancer at the time of surgery and/or patients who had received treatment for distant metastases were excluded. Also, we excluded patients with LLL at baseline.

A retrospective review was conducted on the medical records of all the participating women. Data collected included age, pre-treatment FIGO clinical stage (International Federation of Gynecology and Obstetrics 1995), metastatic status, number of pregnancies and deliveries, post-surgical histopathological classification (International Union Against Cancer, TNM Classification of Malignant Tumours), and pelvic tumor status.

Treatment-related information collected included the date of operation, operative procedures undergone, whether pelvic and para-aortic lymph node dissection or both were carried out, and which preoperative and postoperative adjuvant therapies were administered, including chemotherapy and radiation therapy. We defined preoperative and postoperative adjuvant therapies as those performed within six months before or after the resective operation, respectively.

We identified patients with lymphedema through their medical records or subjective judgment by the patients regarding swelling and heaviness of the legs. LLL was identified via a thorough review of all available records. The study focused only on 'symptomatic' limb lymphedema. It is very likely that some patients in this study had minimal or mild limb lymphedema that would have been detected only by prospective serial limb circumference or volume measurements, which were not undertaken in this retrospective study. The women described symptomatic and persistent swelling in one or both lower limbs, which developed following treatment for her cancer and that could not be attributed to any other cause.

The primary endpoint of this study was the occurrence of LLL. The incidence of lymphedema was tested using Fisher's exact test according to cancer type, in relation to surgical factors that might influence the onset of lymphedema (retroperitoneal approach (opening or closing), omentectomy, enterectomy, hysterectomy, salpingo-oophorectomy, and lymph node dissection), as well as other factors, including patient characteristics and a history of chemotherapy and/or radiotherapy. Logistic regression was used to assess the relationship between lymphedema and radiotherapy. SAS ver. 8.2 (SAS Institute, Cary, NC, USA) was used for the analysis. All reported p-values relate to two-tailed statistical tests.

## Results

Patients' clinical characteristics and treatments received are summarized in Table 1. Almost all patients (98%) underwent pelvic lymph node dissection. More than half the patients underwent bilateral salpingo-oophorectomy.

**Table 1:** Summary of the clinical characteristics of patients involved in this study (N = 694; all female)

	Clinical characteristics	Ovarian cancer		Cervical cancer		Endometrial cancer	
Total no. patients		135		258		301				
Age (years)	Median	51		49		57	
	Range	15–79		23–80		19–80	
FIGO stage (N; %)	I	64	(47.4)	171	(66.3)	196	(65.1)
	II	11	(8.2)	76	(29.5)	27	(9.0)
	III-IV	60	(44.4)	11	(4.3)	75	(24.9)
	Unknown	-	-	-	-	3	(1.0)
Surgical procedure (N; %)	Retroperitoneal						
	Closing	80	(59.3)	184	(71.3)	205	(68.1)
	Opening	32	(23.7)	16	(6.2)	33	(11.0)
	-	23	(17.0)	58	(22.5)	63	(20.9)
	Omentectomy						
	+	87	(64.4)	0	(0.0)	25	(8.3)
	-	48	(35.6)	258	(100.0)	276	(91.7)
	Enterectomy						
	+	9	(6.7)	1	(0.4)	0	(0.0)
	-	126	(93.3)	257	(99.6)	301	(100.0)
	Salpingo-oophorectomy						
	Unilateral	18	(13.3)	34	(13.2)	38	(12.6)
	Bilateral	108	(80.0)	133	(51.6)	203	(67.4)
	-	9	(6.7)	91	(35.3)	60	(19.9)
	Pelvic lymph node dissection						
	+	133	(98.5)	252	(97.7)	295	(98.0)
	-	2	(1.5)	6	(2.3)	6	(2.0)
	Para-aortic lymph node dissection						
	+	71	(52.6)	28	(10.9)	66	(21.9)
	-	64	(47.4)	230	(89.1)	235	(78.1)
	Hysterectomy
	Total	93	(68.9)	6	(2.3)	112	(37.2)
	Modified radical	31	(23.0)	18	(7.0)	150	(49.8)
	Radical	3	(2.2)	230	(89.1)	39	(13.0)
	-	8	(5.9)	4	(1.6)	0	(0.0)
Chemotherapy (N; %)	Neo-adjuvant chemotherapy						
	+	9	(6.7)	20	(7.8)	6	(2.0)
	-	126	(93.3)	238	(92.2)	295	(98.0)
	Adjuvant chemotherapy						
	+	111	(82.2)	62	(24.0)	119	(39.5)
	-	24	(17.8)	196	(76.0)	182	(60.5)
Radiotherapy (N; %)	Pre-operative radiation						
	+	0	(0.0)	6	(2.3)	0	(0.0)
	-	135	(100.0)	252	(97.7)	301	(100.0)
	Post-operative radiation						
	+	1	(0.7)	107	(41.5)	29	(9.6)
	-	134	(99.3)	151	(58.5)	272	(90.4)

Twenty-eight ovarian cancer patients (20.7%), 78 cervical cancer patients (30.2%) and 83 endometrial cancer patients (27.6%) developed lymphedema, with no statistically significant difference between the percentages in the three groups (p = 0.130). The median times from operation to occurrence of LLL were 4.6 months (range: 0.1–40.2 months) for ovarian cancer patients, 4.2 months (range: 0.1–50.7 months) for cervical cancer patients, and 6.8 months (range: 0.1–45.3 months) for endometrial cancer patients (p = 0.258).

The results of the univariate analyses for patients who underwent pelvic lymph node dissection (n = 680) are shown in Table 2. Post-operative radiotherapy (odds ratio (OR): 1.79; 95% confidence interval (CI): 1.20–2.68; p = 0.006) was significantly associated with the occurrence of LLL. Among patients undergoing pelvic lymphadenectomy there was no correlation between any surgical procedure and LLL. Para-aortic lymph node dissection (OR: 1.34; 95% CI: 0.91–19.6; p = 0.158) was not a risk factor for LLL.

**Table 2:** Relationship between factors and lymphedema in ovarian and uterine cancer patients who underwent pelvic lymph node dissection (univariate analysis; N = 680)

Factor	Category	N	No. cases of lymphedema	Lymphedema incidence (%)	p value*
Cancer type	Ovarian	133	28	21.1	
	Cervical	252	75	29.8	
	Endometrial	295	82	27.8	0.175

Age	< 53 years	342	93	27.2	
	≥ 53 years	338	92	27.2	1.000

Retroperitoneal approach**	Closing	457	131	28.7	
	Opening	79	24	30.4	0.789

Omentectomy	-	569	160	28.1	
	+	111	25	22.5	0.245

Enterectomy	-	670	181	27.0	
	+	10	4	40.0	0.473

Salpingo-oophorectomy	-	160	45	28.3	
	Unilateral	87	25	28.7	
	Bilateral	433	115	26.6	0.873

Hysterectomy	-	12	4	33.3	
	Total	206	53	25.7	
	Modified radical	196	51	26.0	
	Radical	266	77	29.0	0.767

Neo-adjuvant chemotherapy	-	645	173	26.8	
	+	35	12	34.3	0.334

Adjuvant chemotherapy	-	393	111	28.2	
	+	287	74	25.8	0.487

Post-operative radiation	-	548	136	24.8	
	+	132	49	37.1	0.006

Para-aortic lymph node dissection	-	516	133	25.8	
	+	164	52	31.7	0.158

Total		680	185	27.2	

* Fisher's exact test

** Except for 144 patients for whom this information was unknown.

Given that different types of cancer require different hysterectomy procedures, we reclassified the patients into those with ovarian or endometrial cancer, who underwent less radical hysterectomy, and those with cervical cancer, who underwent more radical hysterectomy. There was no statistically significant risk factor identified for patients with ovarian or endometrial cancer, but for cervical cancer patients, post-operative radiotherapy was a statistically significant factor for the occurrence of lymphedema (OR: 1.77; 95% CI: 1.03–3.06; p = 0.049).

## Discussion

Lymphedema is generally caused by an obstruction or interruption of the lymphatic system, generally in proximal limb segments at lymph nodes, due to infection, malignancy, or scar tissue [12]. The pelvic and inguinal groups of nodes in the lower limbs are the primary sites of obstruction [13]. When the lymphatic system has been damaged by surgery, its capacity to absorb excess water and cells from the interstitial space is reduced. If the transport capacity of the lymphatic system is so reduced that it cannot manage this increase in lymphatic load, an insufficiency of the lymphatic system will occur [6].

In this study, we found that patients with ovarian cancer had a lower incidence of LLL than did patients with uterine cancer. In a previous study, similar results were found with respect to the incidence of LLL (ovarian cancer: 7.1%; uterine cancer 17.7%; cervical cancer: 17.5%) [14].

Most patients in the present study underwent pelvic lymph node dissection. We found that for ovarian and uterine cancer patients who underwent pelvic lymph node dissection, post-operative radiotherapy was significantly associated with the occurrence of LLL. Post-operative radiotherapy was found to be an independent risk factor for LLL in the present study, confirming the results of previous studies [3,15-17]. Of all patients with ovarian cancer in the present study, only one patient received post-operative radiotherapy, compared with 136 of the uterine cancer patients. The lower incidence of LLL in patients with ovarian cancer might be due to these patients not generally receiving radiation therapy after surgery.

In this study, we found that for patients with ovarian and uterine cancer, para-aortic lymph node dissection is not a significant risk factor for LLL. This finding has a clear biological rationale because it suggests that LLL is caused by pelvic lymph node dissection.

The study design used in the present study had some limitations. Firstly, occurrence of LLL was assessed in a subjective way, and there was no measurement of the severity of the symptoms, nor of the extent of the associated functional impairment. Secondly, we collected no information on the extent of lymph node disease, nor the number of lymph nodes dissected, although previous studies have suggested that the number of lymph nodes surgically removed and the extent of metastatic disease in the lymph nodes are risk factors for lymphedema [10]. Given that we did not collect these data, the relationship between the incidence of LLL and these factors could not be clarified. However, all the institutions that participated in this study specialized in the surgical treatment of ovarian and uterine cancer, and within these institutions twenty or more pelvic and para-aortic lymph nodes are usually removed at operation by gynecological oncologists. Therefore, the patients included in our study can be regarded as having undergone maximum lymph node dissection.

Although at this stage there are no appropriate alternative surgical procedures available to decrease the risk of LLL, our findings have clinical implications with respect to when pelvic lymph node dissection should or should not be performed.

It is important to identify patients at risk of lymphedema early and to begin preventive monitoring and instruction in self-care as soon as possible. The role of nursing in the acute and community care of women at risk of developing LLL includes ensuring that women being discharged are aware of the early signs and symptoms of lower limb lymphedema and how to access qualified, specialized therapists so that effective early management can be initiated [18]. The following parameters are reportedly helpful in the early detection of lymphedema: the ratio of actual to ideal weight, extremity measurements, ability to perform activities of daily living, a history of contributing factors and concurrent medical illnesses (as identified previously for breast cancer patients) [18,19].

## Conclusion

Post-operative radiation was found to be a risk factor for occurrence of LLL among uterine cancer patients who underwent pelvic lymph node dissection. However, there was no association between any surgical procedure and occurrence of lymphedema among patients undergoing pelvic lymphadenectomy. Our findings have a strong underlying biological rationale because they suggest that limb lymphedema is caused by pelvic lymph node dissection.

## Abbreviations

LLL: lower limb lymphedema; OR: odds ratio; CI: confidence interval.

## Competing interests

The authors declare that they have no competing interests.

## Authors' contributions

HT participated in the design of the study, performed the data management and the statistical analysis, and drafted the manuscript. ST participated in the design of the study, performed the statistical analysis, and helped to draft the manuscript. MF participated in the design of the study and helped to draft the manuscript. HS participated in the design and coordination. All authors read and approved the final manuscript.

## Pre-publication history

The pre-publication history for this paper can be accessed here:

http://www.biomedcentral.com/1471-2407/9/47/prepub
